# Climate Change in the Mediterranean Basin (Part II): A Review of Challenges and Uncertainties in Climate Change Modeling and Impact Analyses

**DOI:** 10.1007/s11269-023-03444-w

**Published:** 2023-02-02

**Authors:** L. V. Noto, G. Cipolla, D. Pumo, A. Francipane

**Affiliations:** Dipartimento di Ingegneria, Università degli Studi di Palermo, Viale delle Scienze, Edificio 8, 90128 Palermo, Italy

**Keywords:** Climate change, Mediterranean basin, Hydrological processes, Uncertainty, Water resources

## Abstract

The Mediterranean basin is particularly prone to climate change and vulnerable to its impacts. One of the most relevant consequences of climate change, especially for the southern Mediterranean regions, is certainly water scarcity as result of a reduction of surface runoff and groundwater levels. Despite the progress achieved in recent years in the field of climate change and its impact on water resources, results and outcomes should be treated with due caution since any future climate projection and derived implications are inevitably affected by a certain degree of uncertainty arising from each different stage of the entire modeling chain. This work offers a comprehensive overview of recent works on climate change in the Mediterranean basin, mainly focusing on the last ten years of research. Past and future trends on different components of the hydrological balance are discussed in a companion paper (Noto et al. 2022), while the present paper focuses on the problem of water availability and water scarcity. In addition, the work aims to discuss the most relevant sources of uncertainty related to climate change with the aim to gain awareness of climate change impact studies interpretation and reliability.

## Introduction

As discussed in Noto et al. (2022), the Mediterranean Basin (MB) has experienced, and will likely experience, a reduction in annual rainfall, over most of its areas, and widespread warming, especially during the summer season. The combined effect of the reduction in total annual precipitation and the air warming, which has a direct effect on all the processes involved in the soil water balance, might lead to critical water shortages in several areas of the MB. The most affected countries might be those where water resources are already at a critical level (Tramblay et al. 2020) and their management is still today a critical issue (Zribi et al. 2020). In southern Europe and North Africa, groundwater recharge and soil water content are expected to decline (Braca et al. 2019; Calvache et al. 2020), especially during summer, while river runoff, water levels in lakes, and availability of reservoirs are expected to decrease in many locations of the MB (Braca et al. 2019; Marx et al. 2018; Yeste et al. 2021). The decline in water resources may cause several implications in terms of food and energy security, affecting agriculture sector, with significant losses in crop production (D’Odorico et al. 2013; El-Nashar and Elyamany 2022). As an example, Ortiz-Bobea et al. (2021) evaluated the contribution of the anthropogenic climate change recorded in the period 1961–2015 on global agricultural Total Factor Productivity (TFP), finding out a TPF reduction between -20% and -30% in the MB. These aspects pose serious problems in the expected increase in population, which is set to reach 9.1 billion by 2050, and therefore in the growth of food and energy demand (Leisner 2020).

Starting from the literature review made in the companion paper (Noto et al. 2022), this paper focuses on the main recent works on climate change in the MB across the last ten years of research, limiting the research to works already published in high impact factor journals, selected by using as database Scopus and Google Scholar. In particular the paper focuses on: i) the main challenges that potential future changes may pose in terms of water availability in the MB and ii) the main issues concerning climate change modeling and impact analyses.

Climate change impact assessment usually consists of a modeling chain including the evaluation of emission scenarios, the application of GCMs (Global Circulation Models) and downscaling techniques, the development and application of the hydrological models, often coupled with other impact models (e.g., land use/cover or water use models). Despite the flourishing activity and the progress achieved in this field and its impact on water resources, uncertainty is still an important issue because meteorological and hydrological variables have to be projected some decades into the future (Lempert et al. 1996). Therefore, any result and outcome should be always treated with due caution because of the uncertainty that affects these studies.

Indeed, each stage of the procedure for the assessment of climate impact includes potential sources of uncertainty that may come from measurement errors from instruments, data processing errors, aggregation errors due to incomplete temporal and/or spatial data coverage, unpredictability of future natural processes (e.g., volcanic eruptions, ecosystem dynamics), limited space and time resolution of models, incomplete understanding of some Earth system processes and/or their interaction and feedback, unpredictability of future development of non-climatic (e.g., socio-economic, demographic, technological, and environmental) factors that may affect the emission scenarios and, in turn, local or regional climate.

Climate scenarios selected for a specific impact research should always meet some basic criteria. As an example, they should be representative of the potential range of future regional climate change, and consistent with a broad range of global warming projections based on different Greenhouse Gases (GHGs) emissions scenarios, such as reported in the fifth (AR5) Intergovernmental Panel on Climate Change (IPCC) report, and/or different Shared Socio-economic Pathways (SSPs), such as reported in the sixth (AR6) IPCC report. Moreover, since impact models require, as input, data variables (e.g., precipitation, solar radiation, temperature, humidity, and wind speed) at different temporal (from the annual to the sub-hourly) and spatial (from the global to the plot) scales, climate scenarios should be capable to describe changes in a sufficient number of variables on a spatial and temporal scale. In addition, the choice of the most appropriate hydrological, water management, and system behavior-based models, the adopted method of models’ integration and their parameterization under non-stationary conditions require considerable attention.

The combination of uncertainties coming from the use of different scenarios, models, and analysis techniques at the different stages typically results in a more or less significant overall inaccuracy of the final results (Kim et al. 2019; Lehner et al. 2020; Maher et al. 2021), which has to be carefully considered by policymakers.

The present paper is organized as follows. Section 2 discusses possible problems on future water scarcity in the MB. Section 3 focuses on the main challenges and uncertainties sources in the impact analysis of climate change. Finally, last section reports conclusions of the review study.

## Potential Future Water Scarcity in the MB

Water resource is crucial for the economy of several Mediterranean countries, where rain-fed agriculture accounts for more than 90% of crops in Algeria, Morocco, and Tunisia (Schilling et al. 2020), 57% of crops in Turkey, 64% of crops in Italy, and 56% of crops in Portugal (Jacobsen et al. 2012). According to Jiménez Cisneros et al. (2014), climate change is likely to causing a significant reduction in fresh water availability over the MB, with decreases by 2–15% for 2 °C of warming (Cramer et al. 2018), among the largest in the world, and relevant increases in the length of meteorological dry spells (Kovats et al. 2014; Schleussner et al. 2016) and droughts (Tsanis et al. 2011). A possible decrease in surface runoff, as well as in groundwater levels (Noto et al. 2022), would imply a decline in water supply to natural and artificial reservoirs, which are mainly used for irrigation. Considering the per-capita water availability critical threshold of 1000 m^3^ year^−1^, generally accepted for severe water stress, for some regions already characterized by under-threshold values, such as the southeastern Spain and the southern shores, the per-capita water availability may drop below 500 m^3^ year^−1^ in the next future. Some countries, such as Greece and Turkey, may fall for the first time under critical conditions of water availability by the 2030 (Ludwig et al. 2010).

Analyses of precipitation-related impacts reveal an overall regional reduction in water availability for the Mediterranean, especially in the south and the east. Median reduction will be equal to about 9% (likely range from 4.5% to 15.5%) at 1.5 °C and almost doubled at 2 °C (about 17% with likely range from 8 to 28%). The projected lengthening of regional dry spells, instead, will increase from 7 to 11% (Schleussner et al. 2016).

Over the MB, water demand is already high and may further increase in the future (Fiorillo et al. 2021), especially in North Africa (Droogers et al. 2012), thus affecting water resources availability (Milano et al. 2013). In a study on six basins in southern Europe, located across Portugal, Spain, France, Switzerland, Italy, Greece, Macedonia, Bulgaria, and Turkey, Sordo-Ward et al. (2019) showed how traditional water scarcity problems will be aggravated in the future, with the available resources that will hardly meet the existing water demands in many basins.

In this context, the development of efficient integrated models capable to consider possible future water management strategies becomes a priority to plan efficient adaptation measures.

According to Forzieri et al. (2014), streamflow minima (7 day minimum flows) are projected to decrease up to 40% exclusively due to climate change (Assessment Report 4—A1B scenario over the period 2080–2100) in the for the Iberian Peninsula, Italy, and the Balkan region. Reductions will likely reach -70% considering also increasing water use scenarios, due to drivers such as total population, gross domestic product, electricity production, agricultural production, and technological changes, are included.

In Gomez-Gomez et al. (2022), future water demand scenarios have been generated for the Segura basin (southern Spain) accounting for statistical projections of population and crop water future requirements computed, based on future climate scenarios. In response to a future reduction of mean annual precipitation equal to about -28%, a more significant mean reduction of the available resources (41–59%) was predicted for the period 2071–2100. This was ascribed to a decrease in the mean monthly runoff (from -32% to -67%) and a simultaneous relevant increase in mean monthly water demand from June to February (over 110% for most of the considered ensemble scenarios) and a slight decrease from the remaining months (from -1% to -15%).

Water consumes in the MB are prevalently due to irrigated agriculture, with, on average, about 50% of the total water withdrawal intended for agriculture (Fader et al. 2016), and significant variations over the different Mediterranean sub-regions (e.g., 1.3% in Croatia, 12% in France, 90% in Syria, Egypt, Cyprus, and Greece). As it emerges from the trends and projections discussed in Noto et al. (2022), future climate in this region is expected to shift towards warmer and drier scenarios, also decreasing available groundwater resources (Collins et al. 2013; García-Ruiz et al. 2011; Kovats et al. 2014).

The general increase in water scarcity due to climate change is further exacerbated by the increasing demand for irrigated agriculture to stabilize production and to maintain food security (Albiac et al. 2013; Iglesias et al. 2012). In particular, the projected increment in the irrigation demands for the MB, because of the climate change, is equal to 4% and 18%, for 2 °C and 5 °C warming, respectively, by the end of the century. According to Fader et al. (2016), additional irrigation by 25% in northern and two-fold in south-eastern Mediterranean could be needed.

The analysis of potential problems in future water scarcity should also consider the role of reservoir storage in enhancing resilience to climate change. Some studies have used coupled climatic and system behavior-based models to study the impact of reservoirs, confirming the effective climate change mitigation role that they may have in future (Alimohammadi et al. 2020; He et al. 2020), especially in semiarid regions of the MB (Gorguner and Kavvas 2020; Granados et al. 2021) where reservoir storages could be key infrastructures to overcome variability and to enhance water availability. For instance, Granados et al. (2021), considering 35 different climatic projections for the period 2070–2100, presented an analysis of 16 major Southern European basins investigating possible advantages due to reservoirs under climate change scenarios, concluding that the climate change induced reduction of water availability could be attenuated with increasing storage capacity and a careful management of such water reources.

## Challenges and Uncertainties in Climate Change Modeling and Impact Analyses

Despite many efforts have been recently carried out to quantify climate change impacts on water resources (Kim et al. 2019), the outcomes of such studies should always be read being aware that they are affected by uncertainty due to different sources (Pastén-Zapata et al. 2022). As previously said, uncertainties can propagate through the entire modeling chain (i.e., emission scenario, GCM, downscaling, and impact models), and it is never easy to quantify and reduce uncertainties associated with each source (Chokkavarapu and Mandla 2019). For this reason, climate change impacts should always be supported by opportune sensitivity analyses carried out within a probabilistic framework.

**Fig. 1:**
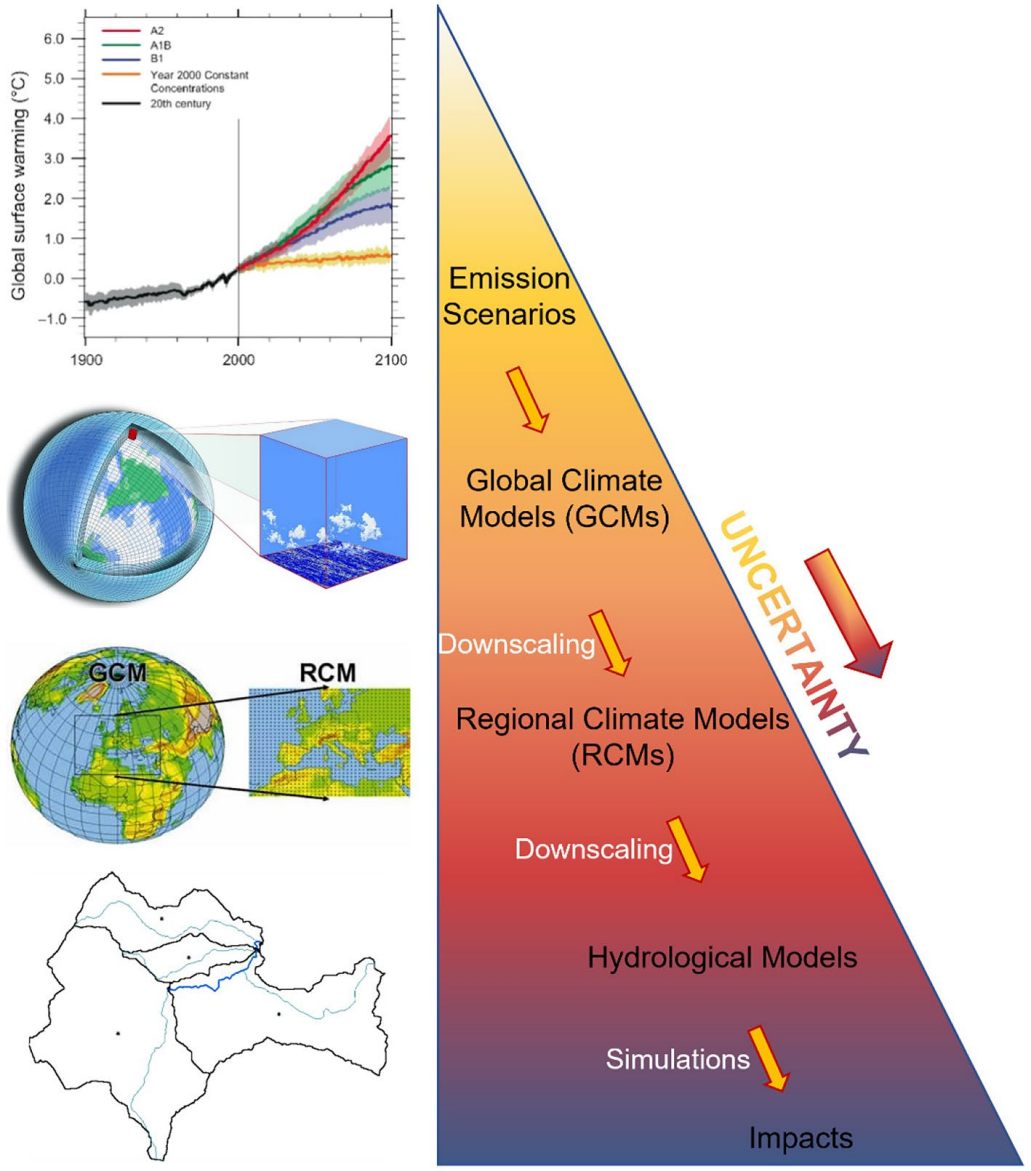
Schematic representation of different sources of uncertainty in climate predictions and impact analyses

The following sections discuss the different sources of uncertainty, schematically represented in Fig. 1, and their influence on the assessment impact of climate change.

### Emission Scenarios

Since 1990 the IPCC has been developing long-term emission scenarios on GHG concentrations for a range of likely futures derived under different assumptions on driving forces such as demographic, technologic, and socio-economic modifications and their combined effects (IPCC 2007). Many of the analysis presented above and in the companion paper (Noto et al. 2022) are usually based on the four Representative Concentration Pathways (RCPs), namely RCP2.6, RCP4.5, RCP6.0, and RCP8.5, developed in the IPCC AR5 and referred to four different approximated level of radiative forcing reached at the year 2100. Lately, nine new scenarios incorporating five Shared Socio-economic Pathways, SSPs (i.e., from SSP1 to SSP5), and seven levels of radiative forcing (i.e., 1.9, 2.6, 3.4, 4.5, 6.0, 7.0, 8.5 W/m^2^) have been developed in the AR6. These are the result of a detailed scenario generation process considering potential future changes of both society and climate to find climate impacts along with possible hypothesized adaptation and mitigation options (O’Neill et al. 2017).

The choice of the most appropriate emission scenario is never trivial because of the many unpredictable variables that should be taken into account (Jose and Dwarakish 2020). Hence, a range of possible scenarios should be considered rather than adopting a single best or average case (Kundzewicz et al. 2018; Vano et al. 2015), especially for long-term impact analyses (Ntegeka et al. 2014; Talchabhadel and Karki 2019). According to Vetter et al. (2017), in fact, the relative importance of the uncertainty in emission scenarios on climate models depends on the temporal horizon of projection.

Obviously, emissions scenarios cannot cover all eventualities and/or predict how the factors that affect GHG and aerosols emissions (e.g., population growth, economic and social development, utilization of carbon-free energy sources and technologies, changes in agricultural practices, land use strategies, technological developments, mitigation policies, etc.) will evolve in the future; this is certainly the largest source of uncertainty in climate modeling. A good example of uncertainties due to the unpredictability of future events is given by two of the most recent events occurred at a global scale: the Coronavirus pandemic (COVID-19) and the conflict between Russia and Ukraine. Because of the population lockdowns, restrictions in movement and reduced energy demand due to the COVID-19 pandemic, global emission levels in 2020 have shown the largest annual decline in history. Le Quere et al. (2020) estimated reductions in daily global fossil CO_2_ emissions of about 17% by early April 2020 compared with mean levels of 2019. Similarly, the outbreak of the conflict between Russia and Ukraine is having a dramatic impact on the exploitation, distribution, and the price of natural resources, such as petroleum and gas. This has having important fallouts, especially on the energy sector, and is leading to relevant changes worldwide in the polity for energy production, trade, and distribution as well as in the use and consumption of different types of energy, with crucial implications on CO_2_ emissions. All these unpredictable factors increase the uncertainties related to the use of the existing emission projections.

### Climate Modeling

Climate system is a complex, non-linear, dynamical system, and understanding the behaviors of its single components does not imply a complete and perfect understanding of the overall behavior.

Knowledge of future climate change comes mostly from GCMs that are numerical models capable to represent physical processes associated with the ocean, land, atmosphere, and cryosphere, as response of the global climate system to increasing GHG concentrations. The GCMs uncertainties mainly come because of an insufficient representation of the climate system, their model structure, and choice of initial conditions used to make simulations (Bennett et al. 2012; Woldemeskel et al. 2014). Indeed, many properties of the processes that take place in atmosphere at a smaller scale than that typical of GCMs need to be averaged over larger scales, to be properly modeled, with a technique known as parameterization. Among these processes, precipitation is perhaps the most important variable subjected to this type of uncertainty. Stephens et al. (2010) described the ability of GCMs to simulate precipitation as ‘dreadful’; precipitation estimates derive from complex processes that are mostly parametrized in atmospheric models owing to their nonlinear nature and multiscale aspects that are still not well known and far from being sufficiently resolved (Tapiador et al. 2019); this aspect represents one of the main challenges in GCM-based impact analyses of climate change.

Other sources of uncertainty related to the use of GCMs are more closely linked to the simulation of various feedback mechanisms concerning, for example, water vapor and warming, clouds and radiation, ocean circulation, etc., which are also the main reason why GCMs may simulate quite different responses to the same forcing.

To assess the uncertainty coming from GCMs, climate model ensembles formed by multiple GCMs are often used (Chen et al. 2011; Knutti et al. 2010; Wang et al. 2020), even though, it might result in large computational costs in the quantification of impacts deriving from climate change. On the other hand, not using all models available might lead to smaller computational costs but to underestimate uncertainty and/or produce a bias in an ensemble prediction (Tebaldi and Knutti 2007).

Nowadays, however, some high-resolution climate models, such as the Convection-Permitting Model (CPM) developed by the UK Met Office (Kendon et al. 2019), can overcome the uncertainty due to above-mentioned parametrization. This model, for example, provides possible future climate scenarios under the RCP8.5 for the UK at a 2.2 km spatial resolution and at the hourly or daily scale. This high spatial resolution makes it possible to characterize local processes, such as convection, influence of mountains or coastlines on the patterns of various climatic variables, that with the coarse resolutions of GCMs (i.e., 60–300 km) or RCMs (i.e., 10–50 km) cannot be properly simulated without parametrization. Despite CPMs may give a much more realistic representation of precipitation compared to coarser resolution climate models, Kendon et al. (2021) highlighted the following deficiencies and key challenges that have to be addressed: i- heavy rainfall tends to be too intense due to convection not being fully resolved at kilometer-scales; ii- inadequate representation of land-surface processes and how they are coupled with the atmosphere at kilometer-scale resolution, with parametrization schemes for sub-kilometer processes that require further developments for use in CPMs; iii- the inclusion of atmosphere–ocean coupling as lateral boundary forcings in CPMs, typically neglected, which could be critical for accurate predictions over some regions; iv- multi-year climate simulations at kilometer-scales are characterized by high computational costs and this may limit the model domain size, the number of ensemble members, and the simulations length.

### Downscaling Climate Modeling

The coupling of GCMs with hydrological models to study the impact of climate change on water resources management, such as streamflow, water content in the soil, or groundwater recharge in a catchment is not trivial, especially because of the different spatial scale of GCMs and hydrological models (Buytaert et al. 2010). Since many physical processes occurring at small spatial scales are very important for many local hydrological processes, a downscaling of GCMs climate projections to regional scale, by for instance statistical or dynamical methods, is necessary (Joseph et al. 2018). Inclusion of downscaling represents another source of model uncertainty (Wootten et al. 2017) in addition to uncertainty coming from GCMs outputs (Wootten et al. 2017; Zhang and Soden 2019).

While statistical downscaling tries to find a data-driven relationship between GCM simulated climate variable and regional scale variable (Chen et al. 2020; Smid and Costa 2018), assuming that this relationship remains stationary over time, thus leading to a misrepresentation of future climate changes (Cannon et al. 2015; Hagemann et al. 2011; Vannitsem 2011), dynamical downscaling uses Regional Climate Models (RCMs), thus being more consistent and physically based but more computationally demanding power (Tang et al. 2016).

Several studies have shown that statistical downscaling methods are often unable to overcome the impacts of circulation biases that are deeply rooted in a GCM's climatology (Addor et al. 2016; Maraun et al. 2017), thus reflecting the circulation biases into other fields (e.g., temperature and rainfall) of regional projections and leading to implausible patterns of future climate change.

Dynamical downscaling models, instead, use lower resolution GCMs as boundary conditions and physical principles to reproduce local climate by means of RCMs, which given the great computational cost, are available only for some regions of the Earth (Chokkavarapu and Mandla 2019). In order to reduce uncertainty in future climate change projections, Diallo et al. (2012) proposed an approach based on multi-model RCM ensembles and multi-projection based on different GCMs to create probability distribution functions of future hydrological variables.

In the last decade, many studies have addressed downscaling as a dominant source of uncertainty (e.g., Dobler et al. 2012; Mandal et al. 2016; Pourmokhtarian et al. 2016), even though they have revealed some critical points. The first issue is that many of these studies focus on impact variables used in hydrology and ecology rather than on the climate variables, relying on the implicit assumption that there is a linear relationship between climate and impact variables. However, since this relationship is often highly nonlinear (e.g., Jin et al. 2005), these results may lead to wrong results. Secondly, most of the prior literature focuses on small regions (e.g., river basins) rather than larger regions, not considering that the conclusions obtained for these small-domain studies may not be transferable to larger regions. The uncertainty linked to downscaling may be overcome by extracting some possible future climate scenarios from high-resolution models, such as the UK-CPM previously described since these can help addressing some local atmospheric processes that affect the patterns of different climatic variables.

### Hydrological and Impact Models

Over decades, ever more sophisticated interpretations have been presented to assess climate change impacts on different hydrological processes and their implications (e.g., floods, droughts, water resources management, etc.).

Hydrological models, often based on conceptual rainfall-runoff bucket approaches, are commonly applied to interpret the output of GCMs or stochastic weather generators, simulating the runoff with different perspectives (i.e., floods, water availability) under a future hypothetical scenario. Although it is commonly assumed that the uncertainties in hydrological modeling can be fully represented by using different hydrological models, in non-stationary conditions, additional uncertainties exist (Lespinas et al. 2014). Uncertainties, for example, may arise from the choice of the hydrological model, the way it is used (parameterization, calibration, validation, consistency of models over potential future climate conditions, etc.), and/or possible deficiencies in the model structure itself. Other uncertainties may arise also from the parameter instability due to the possible changes in the physical catchment characteristics and in the dominant processes (Brigode et al. 2013) and/or from the fact that model parameters are highly dependent on the climatic characteristics of the period used to calibrate the hydrological models (Merz et al. 2011) and cannot be representative of the future period. Moreover, different studies highlight how hydrological models may perform worse under changing climatic conditions, with performances that tend to decline in proportion to long-term changes in climatic variables, such as rainfall and temperature (Broderick et al. 2016; Fowler et al. 2021; Saft et al. 2016). As it emerges from many studies, hydrological model structural deficiencies under changing climate plays a prominent role in the total uncertainty of the whole impact modeling chain (Deb et al. 2019; Fowler et al. 2016; Stephens et al. 2018). With this regard, the impact of the evapotranspiration process conceptualization within the hydrological models on runoff and/or groundwater projections may be extremely relevant, especially for applications in most of the water-controlled regions of the MB (Guo et al. 2017). Her et al. (2019) provides an extensive comparison between uncertainty given by multi-GCMs projections and the one related to hydrological models’ structure and parameters in the hydrological assessment of climate change. The authors found that GCM climate projections are responsible for the highest level of uncertainty within the entire climate-hydrological modeling chain, and the most affected variables are those recognized as rapid hydrological components, such as direct runoff. Uncertainty in hydrological modeling parametrization, instead, plays a relevant role in slow hydrological responses.

Most of the hydrological models used in the impact analysis of climate change adopt conceptual “uncoupled” representations of the soil–vegetation–atmosphere dynamics, relying on the concepts of *potential* (PET) or *reference evapotranspiration* (RET), representative of the *atmospheric evaporative demand* (AED), as surrogate of the *actual evapotranspiration* (AET), which implicitly assume stationary environmental conditions in terms of land use and plant physiology. This is obviously questionable considering the evolving land use and vegetation conditions under a changing climate (e.g., Bosmans et al. 2017; Yin et al. 2017). Indeed, while the projections in AED are expected to follow the predicted increase in temperature, AET is more correlated to the decreasing rainfall. Moreover, evidence of the so-called “evaporation paradox” (Dimitriadou and Nikolakopoulos 2021; Qin et al. 2021) has been reported worldwide, with observed pan evaporation or RET that have decreased along with the continuous increase in temperature over the past decades. All this suggests the use of more integrated approaches to better quantify the surface-atmosphere interactions under climate change (Milly and Dunne 2016; Tramblay et al. 2018). Evapotranspiration process representation in hydrological models should be in fact capable to consider water availability, other than the possible changes in vegetation and land cover/use patterns. Moreover, since uncertainty about how vegetation responds to future increases in CO_2_ and the relative consequences for water fluxes are still questioned (Gerten et al. 2014), another aspect that deserves attention in the model conceptualization of the evapotranspiration process is the way to consider dynamically the effect of CO_2_ concentrations in hydrological models.

Beyond unrealistic simulated evapotranspiration, interactions between surface water and groundwater have been identified as a major contributing factor to non-stationarity in modeled rainfall-runoff relationships. In the MB, the groundwater component has a prominent role to satisfy water demands for agricultural, industrial, and domestic use, especially during drought periods (Carmona et al. 2017; Zhuang et al. 2022). In some cases (e.g., Grigg and Hughes 2018), specific adjustments on the adopted hydrological models were proposed to consider the groundwater impacts on streamflow, while in other cases (e.g., Deb et al. 2019) multi-model frameworks coupling hydrological models with groundwater models were adopted.

With reference to impact analyses, uncertainties are mainly related to the unpredictability of the future and to the inevitable assumptions that must be made for the generation of the various future scenarios of land use/cover, water use and demand, population, urbanization, deforestation, etc. For example, very few studies consider the unpredictable change in occurrence rate of fires, very frequent in MB, and their role in affecting evapotranspiration (Poon and Kinoshita 2018a, b) and control the groundwater and overland flow dynamics. Moreover, many models do not include the possible feedback effects of land cover on the hydrological response of the systems; this is particularly relevant for the MB, which is highly characterized by strong modifications in natural vegetation (Chebli et al. 2018; García-Ruiz and Lana-Renault 2011). With this regard, Pumo et al. (2017) and Arnone et al. (2018) proposed a modeling framework to investigate, at the watershed scale, the potential alterations in the main hydrological components (Pumo et al. 2017) and runoff extremes (Arnone et al. 2018), due to changes in climate and land systems. According to the authors climate and land use interact affecting the hydrological response; as compared to changes in the fraction of impervious areas, climate alterations highlighted more weight in altering all the analyzed hydrological indicators.

Properly taking into consideration reservoir regulations, water diversion, inter-basin water transfers, irrigation, and groundwater withdrawals in future water availability and drought assessments is also important to build a good model framework. All these fundamental factors, which are sometimes found to weaken the relationship between climate variations and water availability (Shah et al. 2021; Wu et al. 2018; Xing et al. 2021; Yang et al. 2020), are closely related to the future trajectories of water uses and demand that are strictly intertwined with different human activities involving a variety of interconnected sectors, such as society, economy, industry, agriculture, and politics.

## Conclusion

The Mediterranean Basin is experiencing several modifications in all the components of the hydrological balance probably due to climate change in addition to the natural inter-annual variability. To this regard, studying trends in water supply is particularly important since water resource determines the economy and the security of several Mediterranean countries. According to the studies explored here, climate change has been leading to a significant reduction in freshwater availability and, in turn, in crop productivity losses, over the MB, contextually to a strong increase in water demand, use and consumption due to population growth and economic development, indicating a higher water stress in the future. In 2015, 196 members of the United Nations Framework Convention on Climate Change negotiated the Paris Agreement. Although this represents an important milestone for fixing sustainable goals, in view of the ongoing climate change in the MB, a big effort is still required to the scientific community and policy makers to promote and implement sustainable solutions to mitigate projected impact on water resources. Innovative research and applied technical solutions, including non-conventional resources, should be explored, also within out-of-the-water-box approaches such as Water-Energy-Food-Ecosystems nexus. Governance should consider the different priorities and needs regarding water and natural resource management within the various areas of the MB, where the differences in water availability and socio-economic aspects between the northern and the southern regions could be amplified by climate change in next future. To mitigate the probable increasing water scarcity, informed and participatory top-down policy-based approaches, involving all the stakeholders, oriented to minimize the anthropogenic impact should be urgently developed by, for instance, reducing the irrigated areas or increasing irrigation efficiency.

The assessment of the impact of climate change requires a complex modeling chain which encompasses emission scenarios, climate models, downscaling and/or bias-correction methods, and hydrological models. Each of these components is characterized by a certain degree of uncertainty that propagates along the entire chain, leading to contrasting and sometimes counterintuitive results. Thus, an important message that arise from the analysis of different experimental studies is that their conclusions must be taken with care, accounting for the different uncertainty sources and trying to understand how they interplay with each other.

Even if some degree of uncertainty may characterize the scientific assessment of climate change impact, it is important to highlight that precautionary approach affirmed by the Principle 15 of the 1992 Rio Declaration reminds us that “*where there are threats of serious or irreversible damage, lack of full scientific certainty shall not be used as a reason for postponing cost-effective measures to prevent environmental degradation*”, thus suggesting to decision-makers needing to apply precaution and the identification of measures aimed to mitigate the different risks related to the climate change impact.

## Data Availability

Not applicable. No datasets were generated or analyzed during the current study.

## References

[CR1] Addor N, Rohrer M, Furrer R, Seibert J (2016) Propagation of biases in climate models from the synoptic to the regional scale: Implications for bias adjustment. J Geophys Res Atmos 121(5):2075–2089

[CR2] Albiac J, Esteban E, Tapia J, Rivas E (2013) Drought in arid and semi-arid regions: A multi-disciplinary and cross-country perspective. Schwabe, K., Albiac, J., Connor, J.D., Hassan, R.M. and Meza González, L. (eds), pp. 323–339, Springer Netherlands, Dordrecht

[CR3] Alimohammadi H, Massah Bavani AR, Roozbahani A (2020) Mitigating the impacts of climate change on the performance of multi-purpose reservoirs by changing the operation policy from SOP to MLDR. Water Resour Manag 34(4):1495–1516

[CR4] Arnone E, Pumo D, Francipane A, La Loggia G, Noto LV (2018) The role of urban growth, climate change, and their interplay in altering runoff extremes. Hydrol Process 32(12):1755–1770

[CR5] Bennett KE, Werner AT, Schnorbus M (2012) Uncertainties in hydrologic and climate change impact analyses in headwater basins of British Columbia. J Clim 25(17):5711–5730

[CR6] Bosmans JH, van Beek LP, Sutanudjaja EH, Bierkens MF (2017) Hydrological impacts of global land cover change and human water use. Hydrol Earth Syst Sci 21(11):5603–5626

[CR7] Braca G, Bussettini M, Ducci D, Lastoria B, Mariani S (2019) Evaluation of national and regional groundwater resources under climate change scenarios using a GIS-based water budget procedure. Rendiconti Lincei-Scienze Fisiche E Naturali 30(1):109–123

[CR8] Brigode P, Oudin L, Perrin C (2013) Hydrological model parameter instability: A source of additional uncertainty in estimating the hydrological impacts of climate change? J Hydrol 476:410–425

[CR9] Broderick C, Matthews T, Wilby RL, Bastola S, Murphy C (2016) Transferability of hydrological models and ensemble averaging methods between contrasting climatic periods. Water Resour Res 52(10):8343–8373

[CR10] Buytaert W, Vuille M, Dewulf A, Urrutia R, Karmalkar A, Célleri R (2010) Uncertainties in climate change projections and regional downscaling in the tropical Andes: implications for water resources management. Hydrol Earth Syst Sci 14(7):1247–1258

[CR11] Calvache ML, Duque C, Pulido-Velazquez D (2020) Summary editorial: Impacts of global change on groundwater in Western Mediterranean countries. Environ Earth Sci 79(24)

[CR12] Cannon AJ, Sobie SR, Murdock TQ (2015) Bias correction of GCM precipitation by quantile mapping: how well do methods preserve changes in quantiles and extremes? J Clim 28(17):6938–6959

[CR13] Carmona M, Máñez Costa M, Andreu J, Pulido-Velazquez M, Haro-Monteagudo D, Lopez-Nicolas A, Cremades R (2017) Assessing the effectiveness of multi-sector partnerships to manage droughts: The case of the Jucar river basin. Earth’s Future 5(7):750–770

[CR14] Chebli Y, Chentouf M, Ozer P, Hornick J-L, Cabaraux J-F (2018) Forest and silvopastoral cover changes and its drivers in northern Morocco. Appl Geogr 101:23–35

[CR15] Chen C, Chen Q, Qin B, Zhao S, Duan Z (2020) Comparison of different methods for spatial downscaling of GPM IMERG V06B satellite precipitation product over a typical arid to semi-arid area. Front Earth Sci 8:536337

[CR16] Chen J, Brissette FP, Leconte R (2011) Uncertainty of downscaling method in quantifying the impact of climate change on hydrology. J Hydrol 401(3–4):190–202

[CR17] Chokkavarapu N, Mandla VR (2019) Comparative study of GCMs, RCMs, downscaling and hydrological models: a review toward future climate change impact estimation. Sn Appl Sci 1(12)

[CR18] Collins M, Knutti R, Arblaster J, Dufresne J-L, Fichefet T, Friedlingstein P, Gao X, Gutowski WJ, Johns T, Krinner G (2013) Climate change 2013-The physical science basis: Contribution of working group I to the fifth assessment report of the intergovernmental panel on climate change, pp. 1029–1136, Cambridge University Press

[CR19] Cramer W, Guiot J, Fader M, Garrabou J, Gattuso J-P, Iglesias A, Lange MA, Lionello P, Llasat MC, Paz S, Peñuelas J, Snoussi M, Toreti A, Tsimplis MN, Xoplaki E (2018) Climate change and interconnected risks to sustainable development in the Mediterranean. Nat Clim Chang 8(11):972–980

[CR20] D’Odorico P, Bhattachan A, Davis KF, Ravi S, Runyan CW (2013) Global desertification: Drivers and feedbacks. Adv Water Resour 51:326–344

[CR21] Deb P, Kiem AS, Willgoose G (2019) A linked surface water-groundwater modelling approach to more realistically simulate rainfall-runoff non-stationarity in semi-arid regions. J Hydrol 575:273–291

[CR22] Diallo I, Sylla MB, Giorgi F, Gaye AT, Camara M (2012) Multimodel GCM-RCM ensemble-based projections of temperature and precipitation over West Africa for the early 21st century. Int J Geophys

[CR23] Dimitriadou S, Nikolakopoulos KG (2021) Evapotranspiration trends and interactions in light of the anthropogenic footprint and the climate crisis: A review. Hydrology 8(4):163

[CR24] Dobler C, Hagemann S, Wilby R, Stötter J (2012) Quantifying different sources of uncertainty in hydrological projections in an Alpine watershed. Hydrol Earth Syst Sci 16(11):4343–4360

[CR25] Droogers P, Immerzeel WW, Terink W, Hoogeveen J, Bierkens MFP, van Beek LPH, Debele B (2012) Water resources trends in Middle East and North Africa towards 2050. Hydrol Earth Syst Sci 16(9):3101–3114

[CR26] El-Nashar W, Elyamany A (2022) Managing risks of climate change on irrigation water in arid regions. Water Resour Manag. 10.1007/s11269-022-03267-1

[CR27] Fader M, Shi S, von Bloh W, Bondeau A, Cramer W (2016) Mediterranean irrigation under climate change: more efficient irrigation needed to compensate for increases in irrigation water requirements. Hydrol Earth Syst Sci 20(2):953–973

[CR28] Fiorillo D, Kapelan Z, Xenochristou M et al (2021) Assessing the impact of climate change on future water demand using weather data. Water Resour Manag 35:1449–1462. 10.1007/s11269-021-02789-4

[CR29] Forzieri G, Feyen L, Rojas R, Flörke M, Wimmer F, Bianchi A (2014) Ensemble projections of future streamflow droughts in Europe. Hydrol Earth Syst Sci 18(1):85–108

[CR30] Fowler KJA, Coxon G, Freer JE, Knoben WJM, Peel MC, Wagener T, Western AW, Woods RA, Zhang L (2021) Towards more realistic runoff projections by removing limits on simulated soil moisture deficit. J Hydrol 600:126505

[CR31] Fowler KJA, Peel MC, Western AW, Zhang L, Peterson TJ (2016) Simulating runoff under changing climatic conditions: Revisiting an apparent deficiency of conceptual rainfall-runoff models. Water Resour Res 52(3):1820–1846

[CR32] García-Ruiz JM, Lana-Renault N (2011) Hydrological and erosive consequences of farmland abandonment in Europe, with special reference to the Mediterranean region – A review. Agr Ecosyst Environ 140(3):317–338

[CR33] García-Ruiz JM, López-Moreno JI, Vicente-Serrano SM, Lasanta–Martínez T, Beguería S (2011) Mediterranean water resources in a global change scenario. Earth Sci Rev 105(3):121–139

[CR34] Gerten D, UK RB, Döll P (2014) Active role of vegetation in altering water flows under climate change. Clim Change

[CR35] Gomez-Gomez J-D-D, Pulido-Velazquez D, Collados-Lara A-J, Fernandez-Chacon F (2022) The impact of climate change scenarios on droughts and their propagation in an arid Mediterranean basin. A useful approach for planning adaptation strategies. Sci Total Environ 820:153128 35041962 10.1016/j.scitotenv.2022.153128

[CR36] Gorguner M, Kavvas ML (2020) Modeling impacts of future climate change on reservoir storages and irrigation water demands in a Mediterranean basin. Sci Total Environ 748:141246 32798863 10.1016/j.scitotenv.2020.141246

[CR37] Granados A, Sordo-Ward A, Paredes-Beltrán B, Garrote L (2021) Exploring the role of reservoir storage in enhancing resilience to climate change in Southern Europe. Water 13(1):85

[CR38] Grigg AH, Hughes JD (2018) Nonstationarity driven by multidecadal change in catchment groundwater storage: A test of modifications to a common rainfall–run-off model. Hydrol Process 32(24):3675–3688

[CR39] Guo D, Westra S, Maier HR (2017) Impact of evapotranspiration process representation on runoff projections from conceptual rainfall-runoff models. Water Resour Res 53(1):435–454

[CR40] Hagemann S, Chen C, Haerter JO, Heinke J, Gerten D, Piani C (2011) Impact of a statistical bias correction on the projected hydrological changes obtained from three GCMs and two hydrology models. J Hydrometeorol 12(4):556–578

[CR41] He S, Guo S, Yang G, Chen K, Liu D, Zhou Y (2020) Optimizing operation rules of cascade reservoirs for adapting climate change. Water Resour Manag 34(1):101–120

[CR42] Her Y, Yoo S-H, Cho J, Hwang S, Jeong J, Seong C (2019) Uncertainty in hydrological analysis of climate change: Multi-parameter vs. multi-GCM ensemble predictions. Sci Rep 9(1):4974 30899064 10.1038/s41598-019-41334-7 PMC6428897

[CR43] Iglesias A, Quiroga S, Moneo M, Garrote L (2012) From climate change impacts to the development of adaptation strategies: Challenges for agriculture in Europe. Clim Change 112(1):143–168

[CR44] IPCC (2007) Climate change 2021: the physical science basis. In: Masson-Delmotte V, Zhai P, Pirani A, Connors SL, Péan C, Berger S, Caud N, Chen Y, Goldfarb L, Gomis MI, Huang M, Leitzell K, Lonnoy E, Matthews JBR, Maycock TK, Waterfield T, Yelekçi O, Yu R, Zhou B (eds) Contribution of working group I to the sixth assessment report of the intergovernmental panel on climate change. Cambridge University Press, Cambridge, United Kingdom and New York, NY, USA, p 2391. 10.1017/9781009157896

[CR45] Jacobsen SE, Jensen CR, Liu F (2012) Improving crop production in the arid Mediterranean climate. Field Crop Res 128:34–47

[CR46] Jiménez Cisneros BE, Oki T, Arnell NW, Benito G, Cogley JG, Doll P, Jiang T, Mwakalila SS (2014) Freshwater resources. In: Field CB, Barros VR, Dokken DJ, Mach KJ, Mastrandrea MD, Bilir TE, Chatterjee M, Ebi KL, Estrada YO, Genova RC, Girma B, Kissel ES, Levy AN, MacCracken S, Mastrandrea PR, White LL (eds) Climate change 2014: impacts, adaptation, and vulnerability. Part A: Global and Sectoral Aspects. Contribution of Working Group II to the Fifth Assessment Report of the Intergovernmental Panel on Climate Change . Cambridge University Press, Cambridge, United Kingdom and New York, NY, USA, p 229–269

[CR47] Jin YH, Kawamura A, Jinno K, Berndtsson R (2005) Nonlinear multivariable analysis of SOI and local precipitation and temperature. Nonlin Processes Geophys 12(1):67–74

[CR48] Jose DM, Dwarakish GS (2020) Uncertainties in predicting impacts of climate change on hydrology in basin scale: a review. Arab J Geosci 13(19):1037

[CR49] Joseph J, Ghosh S, Pathak A, Sahai AK (2018) Hydrologic impacts of climate change: Comparisons between hydrological parameter uncertainty and climate model uncertainty. J Hydrol 566:1–22

[CR50] Kendon EJ, Fosser G, Murphy J, Chan S, Clark R, Harris G, Lock A, Lowe J, Martin G, Pirret J, Roberts N, Sanderson M, Tucker S (2019) UKCP convection-permitting model projections: Science report, p. 153, Met Office Tech Rep. Source: Met Office © Crown Copyright 2019. https://www.metoffice.gov.uk/

[CR51] Kendon EJ, Prein AF, Senior CA, Stirling A (2021) Challenges and outlook for convection-permitting climate modelling. Phil Trans R Soc A 379(2195):20190547. 10.1098/rsta.2019.0547 33641460 10.1098/rsta.2019.0547

[CR52] Kim Y, Ohn I, Lee J-K, Kim Y-O (2019) Generalizing uncertainty decomposition theory in climate change impact assessments. J Hydrol X 3:100024

[CR53] Knutti R, Furrer R, Tebaldi C, Cermak J, Meehl GA (2010) Challenges in combining projections from multiple climate models. J Clim 23(10):2739–2758

[CR54] Kovats RS, Valentini R, Bouwer L, Georgopoulou E, Jacob D, Martin E, Rounsevell M, Soussana J (2014) Climate change 2014: Impacts, adaptation, and vulnerability. Part B Reg Asp Europe 1267–1326

[CR55] Kundzewicz ZW, Krysanova V, Benestad RE, Hov Ø, Piniewski M, Otto IM (2018) Uncertainty in climate change impacts on water resources. Environ Sci Policy 79:1–8

[CR56] Le Quere C, Jackson RB, Jones MW, Smith AJP, Abernethy S, Andrew RM, De-Gol AJ, Willis DR, Shan YL, Canadell OS, Friedlingstein PER, Creutzig EL, Peters E (2020) Temporary reduction in daily global CO2 emissions during the COVID-19 forced confinement. Natu Clim Change 10(7):647-+

[CR57] Lehner F, Deser C, Maher N, Marotzke J, Fischer EM, Brunner L, Knutti R, Hawkins E (2020) Partitioning climate projection uncertainty with multiple large ensembles and CMIP5/6. Earth Syst Dyn 11(2):491–508

[CR58] Leisner CP (2020) Review: Climate change impacts on food security- focus on perennial cropping systems and nutritional value. Plant Sci 293:110412 32081261 10.1016/j.plantsci.2020.110412

[CR59] Lempert RJ, Schlesinger ME, Bankes SC (1996) When we don’t know the costs or the benefits: Adaptive strategies for abating climate change. Clim Change 33(2):235–274

[CR60] Lespinas F, Ludwig W, Heussner S (2014) Hydrological and climatic uncertainties associated with modeling the impact of climate change on water resources of small Mediterranean coastal rivers. J Hydrol 511:403–422

[CR61] Ludwig W, Bouwman A, Dumont E, Lespinas F (2010) Water and nutrient fluxes from major Mediterranean and Black Sea rivers: Past and future trends and their implications for the basin‐scale budgets. Glob Biogeochem Cycles 24(4)

[CR62] Maher N, Power SB, Marotzke J (2021) More accurate quantification of model-to-model agreement in externally forced climatic responses over the coming century. Nat Commun 12(1) 10.1038/s41467-020-20635-w PMC7862648 33542219

[CR63] Mandal S, Breach PA, Simonovic SP (2016) Uncertainty in precipitation projection under changing climate conditions: a regional case study. Am J Clim Chang 5(1):116–132

[CR64] Maraun D, Shepherd TG, Widmann M, Zappa G, Walton D, Gutiérrez JM, Hagemann S, Richter I, Soares PM, Hall A (2017) Towards process-informed bias correction of climate change simulations. Nat Clim Chang 7(11):764–773

[CR65] Marx A, Kumar R, Thober S, Rakovec O, Wanders N, Zink M, Wood EF, Pan M, Sheffield J, Samaniego L (2018) Climate change alters low flows in Europe under global warming of 1.5, 2, and 3 °C. Hydrol. Earth Syst Sci 22(2):1017–1032

[CR66] Merz R, Parajka J, Blöschl G (2011) Time stability of catchment model parameters: Implications for climate impact analyses. Water Resour Res 47(2)

[CR67] Milano M, Ruelland D, Fernandez S, Dezetter A, Fabre J, Servat E, Fritsch J-M, Ardoin-Bardin S, Thivet G (2013) Current state of Mediterranean water resources and future trends under climatic and anthropogenic changes. Hydrol Sci J 58(3):498–518

[CR68] Milly PCD, Dunne KA (2016) Potential evapotranspiration and continental drying. Nat Clim Chang 6(10):946–949

[CR69] Noto LV, Cipolla G, Francipane A, Pumo D (2022) Climate change in the mediterranean basin (part I): Induced alterations on climate forcings and hydrological processes. Water Resour Manag. 10.1007/s11269-022-03400-0 10.1007/s11269-023-03444-w PMC9891896 40478137

[CR70] Ntegeka V, Baguis P, Roulin E, Willems P (2014) Developing tailored climate change scenarios for hydrological impact assessments. J Hydrol 508:307–321

[CR71] O’Neill BC, Kriegler E, Ebi KL, Kemp-Benedict E, Riahi K, Rothman DS, van Ruijven BJ, van Vuuren DP, Birkmann J, Kok K, Levy M, Solecki W (2017) The roads ahead: Narratives for shared socioeconomic pathways describing world futures in the 21st century. Glob Environ Chang 42:169–180

[CR72] Ortiz-Bobea A, Ault TR, Carrillo CM, Chambers RG, Lobell DB (2021) Anthropogenic climate change has slowed global agricultural productivity growth. Nat Clim Chang 11(4):306–312

[CR73] Pastén-Zapata E, Eberhart T, Jensen KH et al (2022) Towards a more robust evaluation of climate model and hydrological impact uncertainties. Water Resour Manag 36:3545–3560. 10.1007/s11269-022-03212-2

[CR74] Poon PK, Kinoshita AM (2018a) Estimating evapotranspiration in a post-fire environment using remote sensing and machine learning. Remote Sens 10(11)

[CR75] Poon PK, Kinoshita AM (2018b) Spatial and temporal evapotranspiration trends after wildfire in semi-arid landscapes. J Hydrol 559:71–83

[CR76] Pourmokhtarian A, Driscoll CT, Campbell JL, Hayhoe K, Stoner AM (2016) The effects of climate downscaling technique and observational data set on modeled ecological responses. Ecol Appl 26(5):1321–1337 27755746 10.1890/15-0745

[CR77] Pumo D, Arnone E, Francipane A, Caracciolo D, Noto LV (2017) Potential implications of climate change and urbanization on watershed hydrology. J Hydrol 554:80–99

[CR78] Qin M, Zhang Y, Wan S, Yue Y, Cheng Y, Zhang B (2021) Impact of climate change on “evaporation paradox” in province of Jiangsu in southeastern China. PLoS ONE 16(2):e0247278 33606798 10.1371/journal.pone.0247278 PMC7895390

[CR79] Saft M, Peel MC, Western AW, Perraud J-M, Zhang L (2016) Bias in streamflow projections due to climate-induced shifts in catchment response. Geophys Res Lett 43(4):1574–1581

[CR80] Schilling J, Hertig E, Tramblay Y, Scheffran J (2020) Climate change vulnerability, water resources and social implications in North Africa. Reg Environ Change 20(1):15

[CR81] Schleussner C-F, Lissner TK, Fischer EM, Wohland J, Perrette M, Golly A, Rogelj J, Childers K, Schewe J, Frieler K (2016) Differential climate impacts for policy-relevant limits to global warming: the case of 1.5 C and 2 C. Earth Syst Dyn 7(2):327–351

[CR82] Shah D, Shah HL, Dave HM, Mishra V (2021) Contrasting influence of human activities on agricultural and hydrological droughts in India. Sci Total Environ 774:144959

[CR83] Smid M, Costa AC (2018) Climate projections and downscaling techniques: a discussion for impact studies in urban systems. Int J Urban Sci 22(3):277–307

[CR84] Sordo-Ward A, Granados A, Iglesias A, Garrote L, Bejarano MD (2019) Adaptation effort and performance of water management strategies to face climate change impacts in six representative basins of Southern Europe. Water 11(5):1078

[CR85] Stephens CM, Johnson FM, Marshall LA (2018) Implications of future climate change for event-based hydrologic models. Adv Water Resour 119:95–110

[CR86] Stephens GL, L'Ecuyer T, Forbes R, Gettelmen A, Golaz J-C, Bodas-Salcedo A, Suzuki K, Gabriel P, Haynes J (2010) Dreary state of precipitation in global models. J Geophys Res Atmos 115(D24)

[CR87] Talchabhadel R, Karki R (2019) Assessing climate boundary shifting under climate change scenarios across Nepal. Environ Monit Assess 191(8):520 31359147 10.1007/s10661-019-7644-4

[CR88] Tang G, Zeng Z, Long D, Guo X, Yong B, Zhang W, Hong Y (2016) Statistical and hydrological comparisons between TRMM and GPM level-3 products over a midlatitude basin: Is day-1 IMERG a good successor for TMPA 3B42V7? J Hydrometeorol 17(1):121–137

[CR89] Tapiador FJ, Roca R, Del Genio A, Dewitte B, Petersen W, Zhang F (2019) Is Precipitation a Good Metric for Model Performance? Bull Am Meteor Soc 100(2):223–233 10.1175/BAMS-D-17-0218.1 PMC6951255 31920206

[CR90] Tebaldi C, Knutti R (2007) The use of the multi-model ensemble in probabilistic climate projections. Phil Trans R Soc A Math Phys Eng Sci 365(1857):2053–2075 10.1098/rsta.2007.2076 17569654

[CR91] Tramblay Y, Jarlan L, Hanich L, Somot S (2018) Future scenarios of surface water resources availability in North African Dams. Water Resour Manage 32(4):1291–1306

[CR92] Tramblay Y, Koutroulis A, Samaniego L, Vicente-Serrano SM, Volaire F, Boone A, Le Page M, Llasat MC, Albergel C, Burak S, Cailleret M, Kalin KC, Davi H, Dupuy JL, Greve P, Grillakis M, Hanich L, Jarlan L, Martin-StPaul N, Martinez-Vilalta J, Mouillot F, Pulido-Velazquez D, Quintana-Segui P, Renard D, Turco M, Turkes M, Trigo R, Vidal JP, Vilagrosa A, Zribi M, Polcher J (2020) Challenges for drought assessment in the Mediterranean region under future climate scenarios. Earth-Sci Rev 210

[CR93] Tsanis IK, Koutroulis AG, Daliakopoulos IN, Jacob D (2011) Severe climate-induced water shortage and extremes in Crete. Clim Change 106(4):667–677

[CR94] Vannitsem S (2011) Bias correction and post-processing under climate change. Nonlinear Process Geophys 18(6):911–924

[CR95] Vano JA, Kim JB, Rupp DE, Mote PW (2015) Selecting climate change scenarios using impact-relevant sensitivities. Geophys Res Lett 42(13):5516–5525

[CR96] Vetter T, Reinhardt J, Flörke M, van Griensven A, Hattermann F, Huang S, Koch H, Pechlivanidis IG, Plötner S, Seidou O, Su B, Vervoort RW, Krysanova V (2017) Evaluation of sources of uncertainty in projected hydrological changes under climate change in 12 large-scale river basins. Clim Change 141(3):419–433

[CR97] Wang H-M, Chen J, Xu C-Y, Zhang J, Chen H (2020) A framework to quantify the uncertainty contribution of GCMs over multiple sources in hydrological impacts of climate change. Earth’s Futur 8(8):e2020EF001602

[CR98] Woldemeskel FM, Sharma A, Sivakumar B, Mehrotra R (2014) A framework to quantify GCM uncertainties for use in impact assessment studies. J Hydrol 519:1453–1465

[CR99] Wootten A, Terando A, Reich BJ, Boyles RP, Semazzi F (2017) Characterizing sources of uncertainty from global climate models and downscaling techniques. J Appl Meteorol Climatol 56(12):3245–3262

[CR100] Wu J, Liu Z, Yao H, Chen X, Chen X, Zheng Y, He Y (2018) Impacts of reservoir operations on multi-scale correlations between hydrological drought and meteorological drought. J Hydrol 563:726–736

[CR101] Xing Z, Ma M, Zhang X, Leng G, Su Z, Lv J, Yu Z, Yi P (2021) Altered drought propagation under the influence of reservoir regulation. J Hydrol 603:127049

[CR102] Yang X, Zhang M, He X, Ren L, Pan M, Yu X, Wei Z, Sheffield J (2020) Contrasting influences of human activities on hydrological drought regimes over china based on high-resolution simulations. Water Resour Res 56(6):e2019WR025843

[CR103] Yeste P, Rosa-Canovas JJ, Romero-Jimenez E, Ojeda MGV, Gamiz-Fortis SR, Castro-Diez Y, Esteban-Parra MJ (2021) Projected hydrologic changes over the north of the Iberian Peninsula using a Euro-CORDEX multi-model ensemble. Sci Total Environ 777 10.1016/j.scitotenv.2021.146126 33684765

[CR104] Yin J, He F, Xiong YJ, Qiu GY (2017) Effects of land use/land cover and climate changes on surface runoff in a semi-humid and semi-arid transition zone in northwest China. Hydrol Earth Syst Sci 21(1):183–196

[CR105] Zhang BS, Soden BJ (2019) Constraining climate model projections of regional precipitation change. Geophys Res Lett 46(17–18):10522–10531

[CR106] Zhuang X, Hao Z, Singh VP, Zhang Y, Feng S, Xu Y, Hao F (2022) Drought propagation under global warming: Characteristics, approaches, processes, and controlling factors. Sci Total Environ 156021 10.1016/j.scitotenv.2022.156021 35588839

[CR107] Zribi M, Brocca L, Molle F, Tramblay Y (2020) Water resources in the Mediterranean region. In: Zribi M, Brocca L, Tramblay Y, Molle F (eds) p xv-xix, Elsevier. ISBN 9780128180860. 10.1016/B978-0-12-818086-0.09990-8

